# Peripheral nerve sheath tumors of the upper extremity and hand in patients with neurofibromatosis type 1: topography of tumors and evaluation of surgical treatment in 62 patients

**DOI:** 10.3205/iprs000117

**Published:** 2017-12-05

**Authors:** Reinhard E. Friedrich, Caroline Diekmeier

**Affiliations:** 1 Department of Oral and Craniomaxillofacial Surgery, Eppendorf University Hospital, University of Hamburg, Hamburg

**Keywords:** neurofibromatosis type 1, upper extremity – surgery, hand – surgery, plexiform neurofibroma, MPNST, peripheral nerve sheath tumor

## Abstract

**Objective:** Neurofibromatosis type 1 (NF1) is an autosomal dominant tumor predisposition syndrome with a tendency to develop peripheral nerve sheath tumors (PNST). Plexiform neurofibromas (PNF) are detected in a high proportion of affected patients. The tumors can lead to severe disfigurement and are classified as precancerous. This study examines the surgical procedures that have been performed on large PNST of the upper limb and hand, and investigates whether a specific distribution pattern of the tumors can be detected in surgically treated cases.

**Methods:** Surgical procedures on the upper extremity and hand performed on patients with NF1 were evaluated at an interval of 25 years (1992–2016). Topography of the tumors was classified according to dermatomes. The number of interventions per patient, duration of operations, and complications of the interventions were registered. An overview of the surgical treatment of PNST of the upper limb and hand was obtained from the literature, with special consideration of the genetic background of treated tumors.

**Results:** One hundred and sixty-three surgical interventions on the upper limb and hand were performed in 62 patients with NF1 for the treatment of large PNST, predominantly PNF (age: mean value: 27.33 years, male: 33, female: 29; right side: 25, left side: 26, bilateral: 7). Surgical procedures lasted an average of 72.47 minutes. In approximately half of the patients, one surgical procedure was sufficient. Duration of stay in hospital was on average 7–11 days. Neurological complications were rarely noted and occurred only temporarily. There were no dermatomes affected by PNF with particular frequency. However, some dermatomes were more often simultaneously affected by a PNF at the same time as others.

**Conclusion:** Although the distribution pattern shows some accumulation of tumor localization, tumors are distributed evenly and show very variable size and extent in individual cases. Surgical treatment of PNF of the upper limb and hand helps alleviate the physical discomfort that these patients have from their disfiguring disease. Repeated interventions are necessary relatively often in order to adapt the tumorous region to the outline of the limb and to improve its function.

## Zusammenfassung

**Einleitung:** Die Neurofibromatose Typ 1 (NF1) ist ein autosomal-dominantes Tumorprädispositionssyndrom mit Präferenz zur Entwicklung von peripheren Nervscheidentumoren (PNST). Plexiforme Neurofibrome (PNF) sind in einem hohen Anteil der betroffenen Patienten nachzuweisen. Die Tumoren können zu schweren Entstellungen führen und gelten als Präkanzerose. Diese Untersuchung geht der Frage nach, welche chirurgischen Maßnahmen bei PNF der oberen Extremität und Hand durchgeführt worden sind und ob sich ein bestimmtes Verteilungsmuster der Tumoren bei chirurgisch behandelten Fällen nachweisen lässt.

**Methoden:** Es wurden in einem Intervall von 25 Jahren (1992–2016) die chirurgischen Eingriffe an oberer Extremität und Hand ausgewertet, die bei Patienten mit NF1 durchgeführt worden waren. Die Topographie der Tumoren wurde nach Dermatomen eingeteilt. Es wurden die Anzahl der Eingriffe je Patient, die Dauer der Operationen und Komplikationen der Eingriffe registriert. Ein Überblick über die Literatur der chirurgischen Behandlung von PNST der oberen Extremität und Hand mit besonderer Berücksichtigung des genetischen Hintergrundes dieser Tumoren wurde angestrebt.

**Ergebnisse:** Es wurden 163 Eingriffe an der oberen Extremität und Hand bei 62 Patienten mit NF1 zur Behandlung von PNST durchgeführt, ganz überwiegend bei PNF (Alter: Mittelwert: 27,33 Jahre; männlich: 33, weiblich: 29; Lokalisation: rechts/25, links/26, beidseits/7). Der operative Eingriff dauerte im Mittel 72,47 Minuten. In ca. der Hälfte der Patienten genügte ein operativer Eingriff. Neurologische Komplikationen traten sehr selten auf und waren nicht von Dauer. Die Dauer des stationären Aufenthaltes währte ca. 7–11 Tage.

**Schlussfolgerung:** Das Verteilungsmuster zeigt zwar einige Häufungen von Tumorlokalisationen an, doch sind die Tumoren insgesamt gleichmäßig über die Extremität verteilt und im Einzelfall sehr variabel in Größe und Ausdehnung. Die chirurgische Behandlung von PNF der oberen Extremität und Hand trägt zur Linderung der körperlichen Beschwerden bei, die diese Patienten von ihrer entstellenden Erkrankung haben. Wiederholte Eingriffe sind relativ häufig notwendig, um die tumoröse Region dem Umriss der Extremität anzugleichen und die Funktion der Extremität zu verbessern.

## Introduction

Neurofibromatosis type 1 (NF1) is a human autosomal dominant transmitted hereditary disorder which occurs with relative frequency and carries an increased risk of developing tumors [1]. NF1 patients show a particular tendency to develop peripheral nerve sheath tumors (PNST), termed neurofibroma [2]. The most frequent PNST in NF1 is dermal/cutaneous neurofibroma (CNF) [3]. This type of tumor is limited to a size of up to a few centimeters in diameter and does not grow into deeper body strata. CNF may develop sporadically or in small numbers, but can also occur in the hundreds all over the integument [3]. CNF usually develop after puberty and predominantly are an esthetic problem for affected individuals. On the other hand, some neurofibroma arise from nerve trunks, are large in size and often extend far into several body regions. These tumors are very likely congenital in origin and are termed “plexiform neurofibroma” (PNF) with respect to the growth pattern of intertwined tumorous nerves [3]. PNF only very rarely develop in an individual without diagnosis of NF1 and this isolated finding may represent a segmental type of the disease [4], [5], [6]. PNF can cause severe esthetic *and* functional problems [7], [8]. Furthermore, PNF is regarded as a precancerous lesion [9], [10]. Surgery is the most effective measure to treat patients with PNF [11], [12]. However, due to the large extent of many PNF, complete resection of these lesions is hardly feasible [13] and extensive surgery may cause severe iatrogenic damage [14]. Furthermore, PNF may develop associated with diffuse enlargement of the affected body region. This phenotype is described as “elephantiasis neuro(fibro)matosa” [15]. This denomination illustrates a visible local, disproportional malformation in the form of enlargement of a body region by analogy with a body part of another species, the latter perceived as of natural size and proportion. However, this descriptive diagnostic term provides insufficient information about which tissue has proliferated so enormously [16]. Alternatively, enormous tumors are also termed “massive soft tissue neurofibroma” [3]. This tumor type appears to be restricted to generalized or mosaic NF1 and is said to rarely develop into malignant peripheral nerve sheath tumor (MPNST) [3].

PNF in association with NF1 may develop with variable location and size in the upper extremity and hand, from childhood [17], [18]. For a long time, many reports have been published on the treatment of PNF in NF1 of the upper extremity and hand, most delivered as case reports [19], [20]. Some reports detail surgical experiences on small series of NF1 patients affected by large PNF, or present reviews based on case reports distributed in the literature [13], [15]. Larger series on surgical treatment for NF1-associated PNST of the upper extremity and hand are rare [11], [19], [21], [22], [23]. This report describes more than 20 years of personal experience in the treatment of PNST of NF1-affected patients and will focus on the analysis on PNST arising in the upper extremity and hand.

## Material and methods

Sixty-two patients with PNF of the upper extremity and hand were treated by the senior author over a period of 25 years (1992–2016). Inclusion criteria for this study were diagnosis of PNF or diffuse neurofibroma of the upper extremity and hand. In all cases a surgical procedure for large PNST had taken place in the region of interest. CNF of the region of interest were also recorded in patients who were surgically treated for both types of tumor in this region but were not evaluated further.

All patients were diagnosed as being affected by NF1 according to the updated World Health Organization (WHO) diagnostic criteria [1]. The female to male ratio was 29/33. Retrospective analysis was based on the patients’ medical reports including the operation report for all 62 patients, histology reports, imaging findings (magnetic resonance imaging (MRI) [24], [25], [26], computed tomograms (CT), plain radiographs of the upper extremity and hand, B-scan ultrasound imaging of the region of interest), registration of hospital stay duration, complications during or following surgical procedures and photography of the affected body regions prior to, during and after surgical intervention. Photograph series were incomplete in 12 patients, but the extent of tumors could be derived from the operation report and from medical reports detailing the CT or MRI imaging of the region of interest.

**Figure 1:**
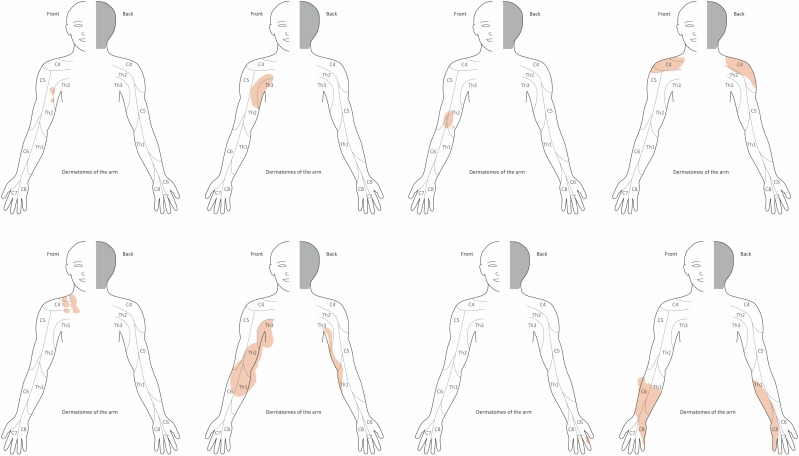
Schematic drawing of dermatomes of the upper limb and hand used for plotting the extent of PNF (illustrated for right arm), exemplified in 8 individuals: areas of tumor affecting the skin (diffuse neurofibroma) or nodular PNF located below the skin are marked in blue. Tumor expansion can continuously infiltrate a skin region without clear reference to one or more dermatomes. Tumors with a smaller area of infiltrated skin are more likely to lie within a dermatome. Some tumors are so large that they alter the contour of the limb. In these areas the color marking of the tumor extension extends beyond the outline of the limb. This simplified schematic drawing does not take into account the highly variable sensory skin innervation of the upper limb as described in detail in anatomical studies [102].

Topography of the tumors was described according to dermatomes [27]. Tumor topography of PNF with respect to dermatomes was assessed using the photographic documentation. For the analysis, the visible tumor of the arms and/or hands was assigned to the dermatomes of that limb. There are some differences in the designation of human dermatomes by authors working in the fields of human topographical anatomy. We decided to include C4 to Th3 dermatomes in our study because several tumors extended to the shoulder and axillary region. Here, Th3 can extend to the upper extremity side of the axilla (Figure 1 (Fig. 1)). It was not necessary for the tumor to have infiltrated the entire dermatome to be assigned to it; the rule was partial infiltration of the dermatome by the visible tumor. It was also the rule that tumors expanding plaque-like in a horizontal (circular) direction perpendicular to the limb’s long axis or stretched along the course of the limb were both usually recorded as tumors extending over several dermatomes. Therefore, the analysis accounted for the possibility that tumors could affect mutually adjacent dermatomes.

Complications of PNF surgery included hematoma that caused a further intervention and neurologic deficits. Oozing blood from the wound drainage during the first days after surgery was not regarded as a complication. A diffuse PNF is strongly vascularized and has a bleeding tendency similar to a hemangioma. Therefore, moderate postoperative bleeding from the operative area during the first days after the surgery is not a rarity. Drainage was regularly applied to allow discharge of oozing blood in order to prevent hematoma. These measures were combined with a pressure bandage for several days. All surgical reports and medical records were checked for blood transfusions in every patient. Neurological deficits of sensitive or motor nerves were also registered as complications. During all surgical procedures, the function of tumor-affected nerves was tested with a nerve stimulator.

The medical history of one of the patients in this group has been described recently [10]. This report discussed in detail the difficult interpretation of sectional and functional imaging to assess tumor biology in PNF, in particular standardized uptake values (SUV) in positron emission tomography (PET) [12]. The history and therapy of one further pediatric patient has been used to illustrate our concept of treating *superficial* PNF with limited extent early in life in order to achieve complete tumor removal in this operationally favorable situation [24]. Initial surgical results of tumor debulking procedures in a third patient have already been presented [7] and will here be re-assessed in long-term follow-up.

### Statistics

For statistical analysis of our findings, gender, age, and location of the tumor (PNF) were collected. For data analysis, metric variables with mean value and standard deviation as well as categorical variables with absolute and relative frequencies were determined. Statistical analysis included the use of student’s t-test and Pearson chi-square test where appropriate.

### Ethics

The investigation was approved by the University Hospital Authority Board as a prerequisite to achieve the doctoral degree at the Faculty of Medicine of the University of Hamburg (CD). All patients had given informed consent for scientific investigation of medical findings. For this investigation, no ethics vote was needed.

## Results

Most PNF developed on one side of the body (right: 25, left: 26). However, bilateral removal of upper extremity PNF was performed in 7 cases (incomplete data: 4).

With the exception of 2 patients, the tumors in all other patients were PNF and showed benign tumor biology during the observation period. One patient who had developed an MPNST of the left upper arm and axilla was treated. This patient had a history of MPNST in the distal region of the lower arm on the same side some years before and this first tumor had likely originated from a PNF. The second patient with MPNST was admitted to hospital for palliative treatment of advanced stage disease, and was treated for temporary coverage of an ulcerating tumor. History of MPNST in other body regions was noted in 4 patients.

About half of the patients also had ablative surgery for CNF of other body regions. However, extensive tumors were only rarely seen outside the upper extremity and would have given the presumption of another PNF or “elephantiasis”. Results are summarized in Table 1 (Tab. 1).

**Figure 2:**
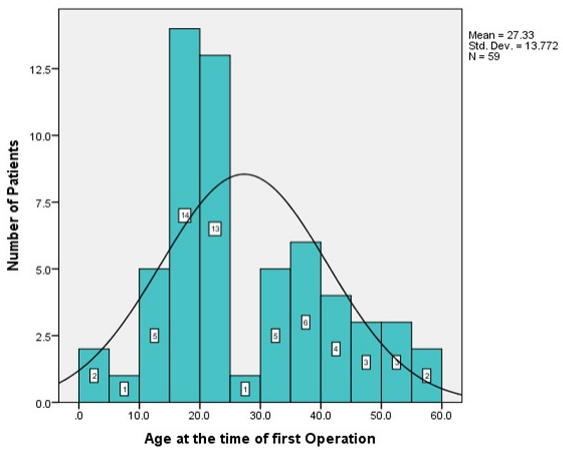
Age of patients at the time of first surgical intervention

**Figure 3:**
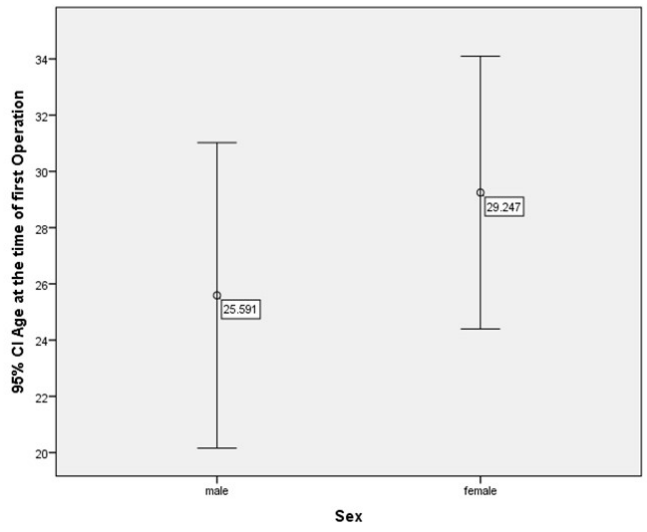
Age of patients at the time of first surgical intervention with respect to gender (CI=confidence interval)

At the time of first operation for PNF in the region of interest, patients were predominantly in the range 15 to 25 years old (males: 3.1 years to 59.6 years; females: 9.4 years to 53.5 years) (Figure 2 (Fig. 2)). The age of the patients at the time of first operation was recorded with respect to gender (Figure 3 (Fig. 3)). The differences in age achieved no statistically significant level (p=0.313, t-test).

**Figure 4:**
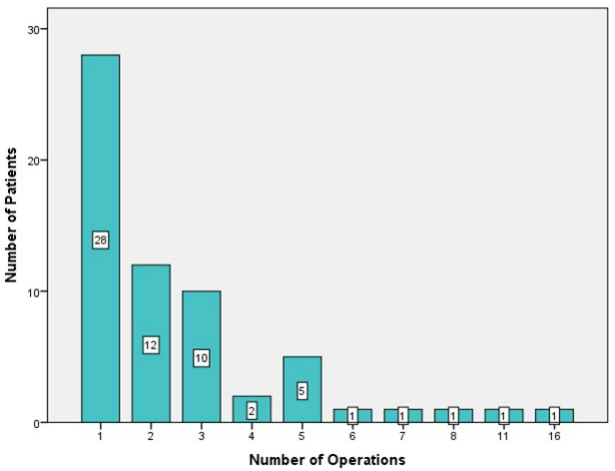
Number of surgical interventions per patient

In approximately half of the patients, one surgical procedure was sufficient to alleviate symptoms (Figure 4 (Fig. 4)). The number of complications related to surgical intervention was low (Table 2 (Tab. 2)).

**Figure 5:**
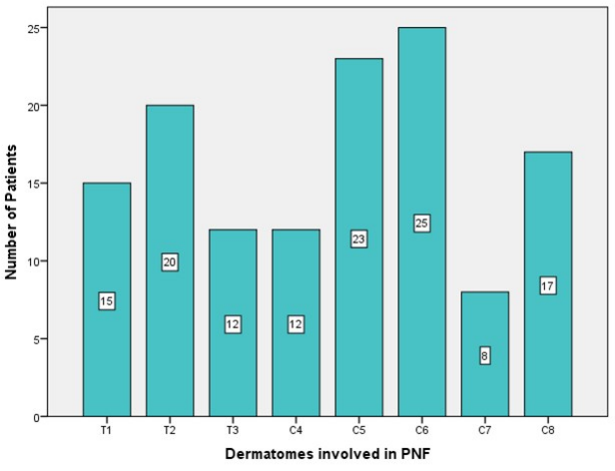
Number of patients related to the respective dermatome of the study region

**Figure 6:**
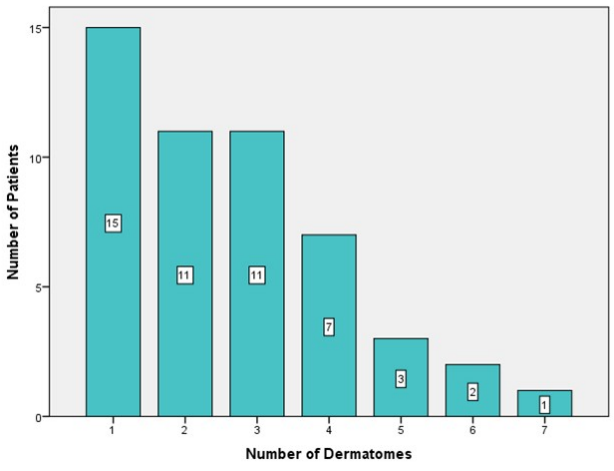
Number of dermatomes per patient affected by tumors/surgical interventions

An attempt was made to assign the extent of the tumors to the respective dermatome of the upper limb and hand. No specific pattern of tumor localization or tumor spread was seen on making this assignment (Figure 5 (Fig. 5)). Fifteen patients (30%) showed one PNF-affected dermatome. Eleven patients (22% each) showed PNF in 2 or 3 dermatomes, respectively. Seven patients (14%) were affected with a PNF in 4 dermatomes, 3 patients (6%) in 5, 3 (4%) in 6, and 1 patient (2%) even in 7 dermatomes (Figure 6 (Fig. 6)).

**Figure 7:**
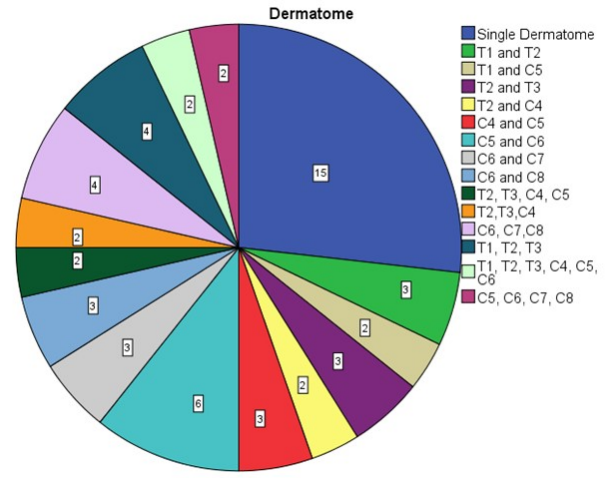
Illustration of the number of dermatomes per patient affected by tumors/surgical interventions

The tumors were widely distributed over the dermatomes to varying degrees (Figure 7 (Fig. 7)). 

**Figure 8:**
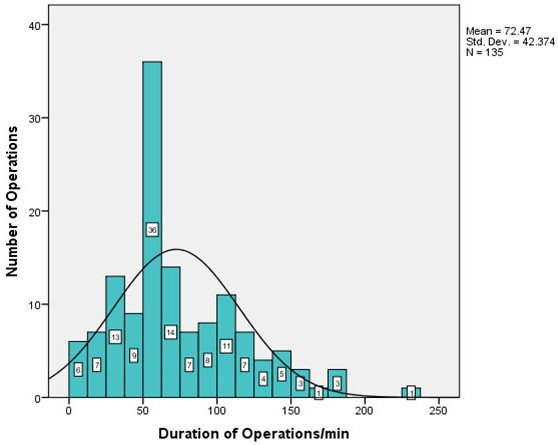
Duration of surgical interventions: resection of peripheral nerve sheath tumors and contouring of the upper limb and hand

**Figure 9:**
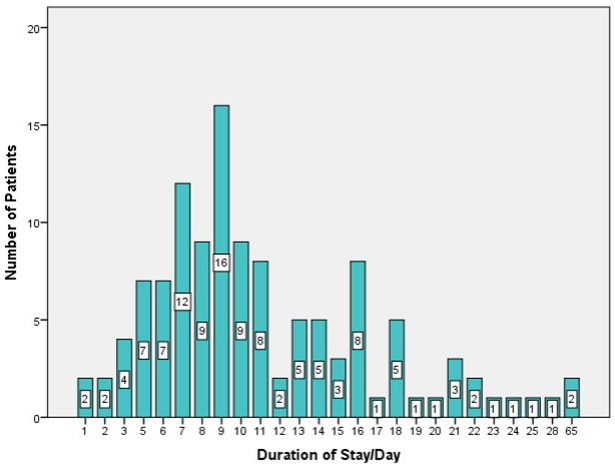
Duration of stay in hospital following surgery for peripheral nerve sheath tumors of the upper limb and hand

**Figure 10:**
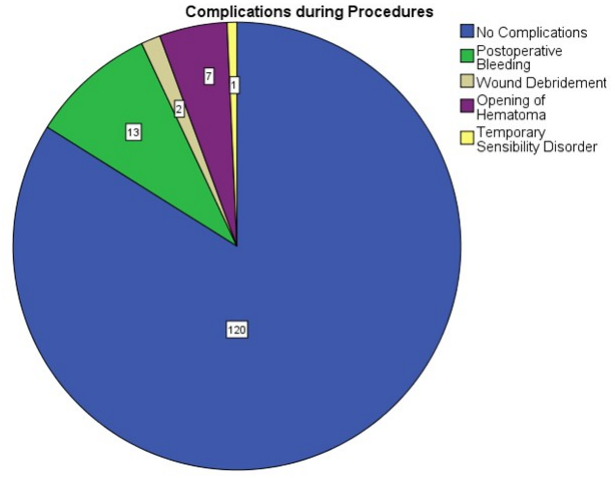
Complications following surgery for peripheral nerve sheath tumors of the upper limb and hand

Mean duration of surgery was 72.47 minutes (Figure 8 (Fig. 8)). However, duration of surgery varied considerably and strongly depended on the extent and volume of the tumor, topography of affected nerves and the unpredictable, usually very lengthy, bleeding time. As a rule, the most extensive bleeding occurred during first intervention or in recurrent interventions after long intervals without surgical exploration. Duration of stay in hospital was usually about 7 to 11 days (54 of 118 procedures), but a detailed listing of hospital stay discloses a substantial proportion of procedures having required a longer period of wound care in treated patients (Figure 9 (Fig. 9) and Figure 10 (Fig. 10), Table 2 (Tab. 2)). Prolonged duration of stay in hospital was predominantly caused by delayed wound healing in extensive diffuse PNF and in cases of elephantiasis. However, in no case was the existing integument resected so far that primary wound closure was no longer possible. Oozing blood was judged a normal finding after debulking procedures in NF1. Indeed, suction drainage was regularly applied in order to allow rapid discharge of oozing blood. Alternatively, a Penrose drain was implemented to prevent development of hematoma. This latter procedure became the preferred drainage method over the years. Allowing slight hemorrhage from the wide and deep wound areas through the silicone tube in combination with a compression bandage has proven to be the most reliable postoperative method of wound care. Indeed, blood administration after surgery was necessary in only 3 patients (2x6 units each for palliative surgery in metastatic MPNST of the upper extremity and trunk; 4 units each in a further 2 patients who had developed hematoma following extensive debulking procedures). A compression bandage was always applied to the operation field for several days after tumor reduction. Some patients refused this aid to bleeding control and consequently had to face complications even several days after surgical intervention. In all cases the skin has closed again after the procedure.

Temporary disorders of epicritic sensibility in the surgical operation area occurred postoperatively and declined in trend during the observation period. However, the detection of motor activity in some tumorous nerves caused us to spare these regions from resection. None of the patients experienced permanent motor nerve deficiency following surgical intervention.

**Figure 11:**
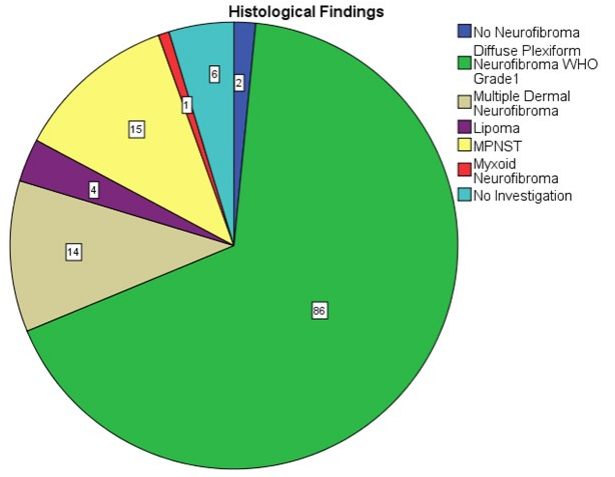
Histology of peripheral nerve sheath tumors of the upper limb and hand

Resection specimens were investigated in the neuropathology department. The vast majority of tumors were classified as diffuse and/or plexiform neurofibroma (Figure 11 (Fig. 11)). The relatively large number of MPNST surgical measures is explained by the multiplicity of palliative interventions in the one case with MPNST and several biopsies that were taken in patients suspected of having advanced stage disease (Table 3 (Tab. 3)).

### Dermatomes

Tumors were assigned to dermatomes of the upper limb and shoulder in 62 patients (dermatomes: C4–C8 and T1–T3) (Figure 5 (Fig. 5), Figure 6 (Fig. 6), Figure 7 (Fig. 7)). Tumors affected 132 dermatomes in 62 patients. Twenty-five patients (18.9%) showed a PNF in dermatome C6, 23 patients (17.4%) in C5, 20 patients (15.2%) in Th2, 17 patients (12.9%) in C8, 15 patients (11.4%) in Th1, 12 patients each (9.1%) in Th3 and C4 and 8 patients (6.1%) in C7.

Associations of tumor-affected dermatomes were further analyzed (Pearson chi-square test, phi coefficient) (Table 4 (Tab. 4)). Dermatomes T2 and T3 showed a strong association (Pearson chi-square=23.68, p<0.001, phi=0.688). A strong correlation was further calculated for simultaneous development of PNF in dermatomes T2 and C4 (Pearson chi-square=12.35, p<0.001, phi=0.497) and C6 and C7 (Pearson chi-square=9.524, p=0.002, phi=0.436). All other correlations of upper limb dermatomes showed weaker correlations of PNF growth (phi<0.393). 

In a further step, it was determined which constellations of jointly affected dermatomes existed. This situation occurred in 56 dermatomes. In 15 patients, only one dermatome was affected. The most frequent finding was synchronous tumor in dermatomes C5 and C6 (6 patients, 10.7%). In 4 patients, dermatomes C6, C7, C8 or Th1, Th2, Th3 were affected by a PNF (7.1% each). Th1 and Th2, Th2 and Th3, C4 and C5, C6 and C7, and C6 and C8 were affected in 3 patients (5.4% each). Six other constellations of affected dermatomes are summarized in Figure 6B (Fig. 6) (2 patients, 3.6% each).

**Figure 12:**
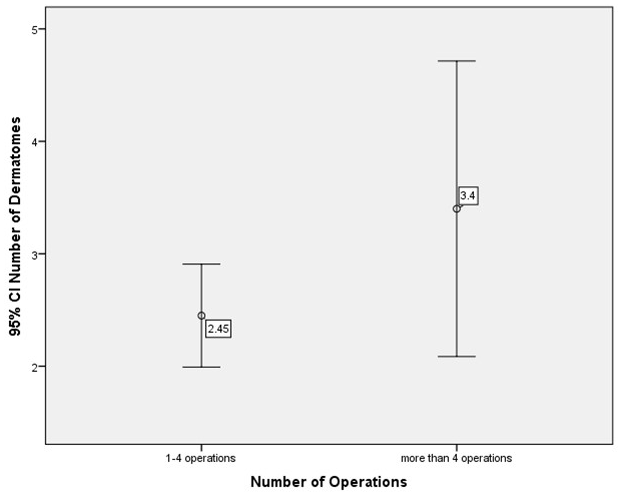
Number of surgical interventions of the upper limb and hand (t-test)

Finally, subdivision of the study group was performed with reference to number of operations. Group 1 showed 1 to a maximum of 4 operations of the upper limb per patient, group 2 more than 4 operations. Comparison of the number of dermatomes with PNF showed that, on average, one more dermatome was affected by PNF in the second group than in the first group. However, this difference in tumor extent had no statistically significant effect on the number of surgical interventions (p=0.083, t-test) (Figure 12 (Fig. 12)).

### Indications for surgical measures 

From a review of the surgical measures, the following indications for surgical interventions for nerve sheath tumors of plexiform/diffuse type in NF1 are given, and are illustrated by individual cases:

#### 1. Exploration/biopsy

**Figure 13:**
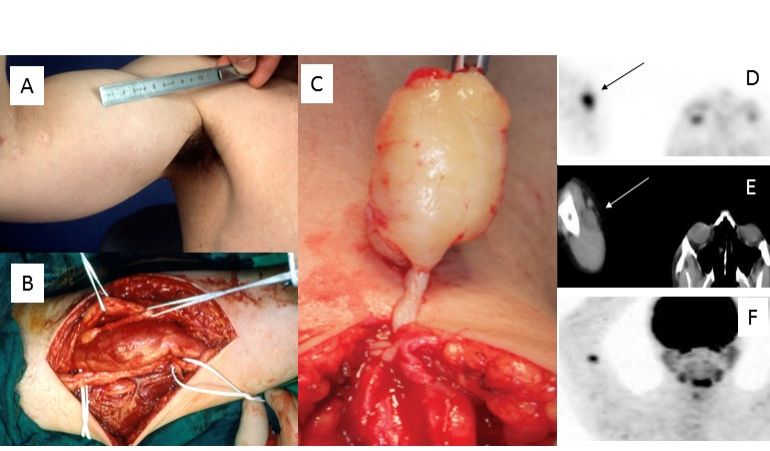
A,B. Exploration of upper extremity for painful multiple plexiform neurofibroma. C–F. Excision of plexiform neurofibroma with elevated standardized uptake value in positron emission tomography in a patient with a history of malignant peripheral nerve sheath tumor

Surgical exploration of space-occupying lesions has to be considered in some individuals with NF1. Rapidly enlarging mass and/or an unusual or novel, long-lasting pain are the predominant findings leading to the decision to explore the affected region (Figure 13A,B (Fig. 13)) [28].

Advanced imaging techniques, e.g. MRI [29] or PET [30], support differentiation between benign and malignant lesions. However, the sensitivity and specificity of the imaging techniques cannot provide complete diagnostic safety in every single case (Figure 13C–F (Fig. 13)). These diagnostic measures cannot yet be taken by the practitioner as true indicators of tumor biology to decide with certainty between a necessity for treatment and a wait-and-see policy. In particular, history of malignant tumor in an NF1-affected individual will very likely support the decision for surgical exploration even if imaging findings do not indicate a malignant transfer of tissues (Figure 13C–F (Fig. 13)).

Furthermore, differential diagnosis of nerve sheath tumors should be considered in space-occupying lesions of the upper extremity and hand in NF1-affected individuals [18], [31], [32].

#### 2. Nodular PNF (localized intraneural neurofibroma)

Nodular PNF usually originate from larger nerves or nerve fascicles. These tumors often are situated in deeper layers of the body. However, tumor growth can cause nerves lying deeper in the body to penetrate subcutaneous tissue with their side facing the body’s surface. Tumors may develop as solitary lesions (Figure 13C–F (Fig. 13)) or affect multiple nerves (Figure 13A,B (Fig. 13)). This tumor type has a higher risk of malignant degeneration if situated in deeper body regions [33]. However, nodular tumors of more superficial body layers are well known in NF1. The complete removal of this type of tumor in any case means resection of the entire tumorous nerve because the PNF infiltrates the single, often extremely enlarged, nerve completely, although inhomogeneously. Resectability of a nodular PNF is therefore based on the extent and function of the tumor nerve (Figure 13A,B (Fig. 13)) and the presumed local tumor biology [34], [35]. Maintenance of nerve continuity in surgical procedures for PNF is identical to that for incomplete tumor resection [3].

#### 3. Superficial PNF

The extent of superficial (diffuse) PNF may vary widely [36], [37], [38]. Since the definition of this tumor type implies that the neoplasm is restricted to the skin, that is, there is no infiltration of the musculature, it is possible to completely remove the tumor if a sufficient and adequate donor region is available to cover the defect [24]. However, in the case of extensive involvement of the body region, this surgical, rehabilitative concept cannot be implemented. The alternative of complete resection and coverage with skin grafts is associated with the risk of necrosis and scarring. Vascularized transplants are a proven alternative for defect coverage in NF1 [39], but they require acceptance of morbidity of the donor region.

From our own experience, contouring of the limb with thinned tumor-infiltrated skin is a gentle alternative. However, this method often requires repeated surgical corrections because:

1. the resected tumor volume is smaller than in radical resection so that further surgical treatment is required and
2. the outline of the surgically treated limb is difficult to estimate due to lack of elasticity of the residual tumorous soft tissue and unpredictable skin sagging following the decrease in swelling after surgery.

**Figure 14:**
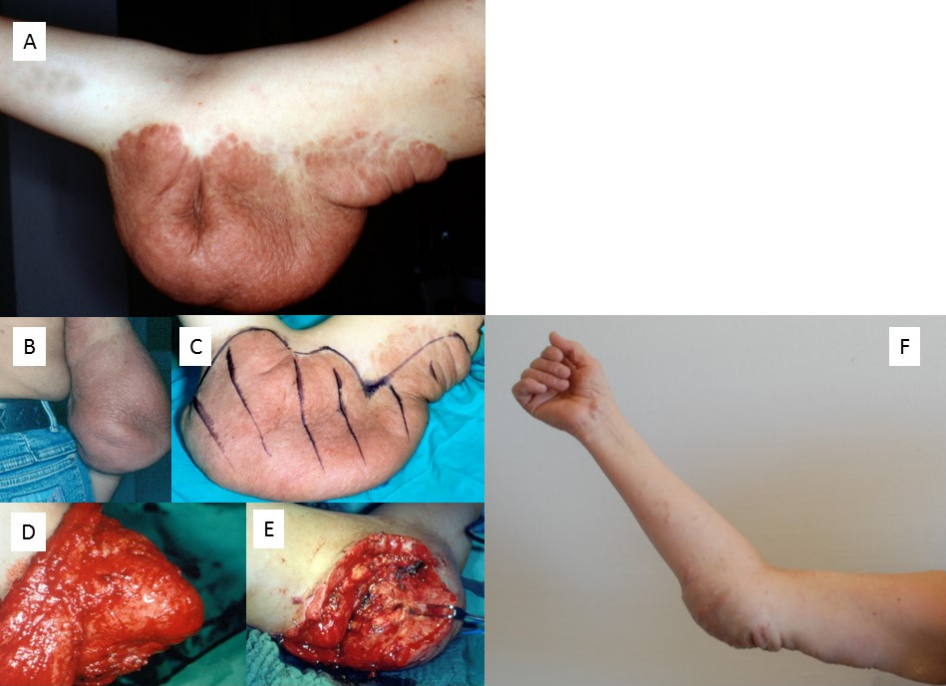
A–E. Debulking of large plexiform/diffuse neurofibroma of the elbow (patient at age 21 years). F. Photograph taken at the age of 41 years, 20 years after first debulking procedure. In the meantime, small corrective operations had been carried out. The result of the initial tumor reduction has remained constant over the decades. Restriction in stretching the arm at the elbow has existed since early childhood and was not influenced by the surgical interventions.

Skin fed via the diffuse PNF cannot be mobilized widely without affecting blood flow, so that rotating lobes are difficult to perform. The wound sutures should not be pulled too tight because the tumorous area reacts to the surgical procedure with considerable swelling which can cause a dehiscence of the wound edges by expansion of the PNF and consecutive delayed wound healing. An illustrative case is shown in Figure 14 (Fig. 14).

#### 4. Invasive PNF

**Figure 15:**
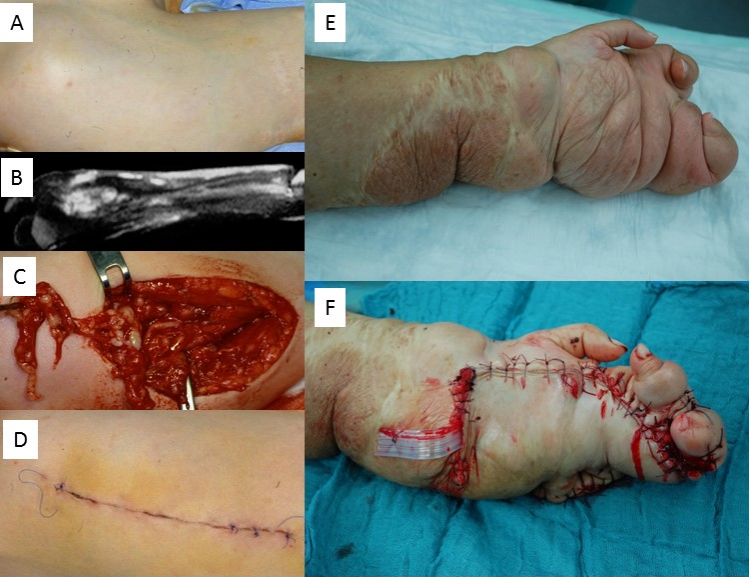
A–D. Resection of plexiform neurofibroma of the right upper extremity in a patient with NF1, who was very sensitive to pain in this area. A. In this case, the effect of the invasive tumor on the skin is hardly visible. Palpation shows only the superficial portions of the nodular tumors (“bag of worms”). Skin pigmentation in this area is hardly changed. B. Magnetic resonance image indicates a nodular tumor and hyperintense skin covering the tumor-affected region. C. Intraoperative situs reveals a dense conglomerate of enlarged and tumorous peripheral nerves. D. Wound closure by primary intention. E. Female patient with NF1 and history of multiple surgical interventions of the left upper extremity. F. Debulking in the palm region was carried out in order to improve gripping function of the remaining fingers.

Invasive PNF is defined as a nerve sheath tumor invading the surroundings without respecting the integrity of adjacent tissues and organs [25], [26]. This tumor is best imaged using MRI, and usually a greater body region is affected. Definition of organs is severely hampered or impossible in these tumor regions. A combination of invasive PNF and localized nodular tumors inside the affected region may occur, as well as the affected skin region adopting the characteristics of superficial PNF covering an invasive PNF [26]. In these patients, the surgical objectives are often limited to improvements in the outline of the body without affecting the tumor load of deeper body regions (Figure 15A–D (Fig. 15)).

An important distinction in this patient group is limitation of tumor growth to the soft tissue or simultaneous tumor-associated alteration of soft tissue and bone [40]. Indeed, the same tumor type can be associated with impressive skeletal alterations, such as localized bony overgrowth, bone deformity or osteolysis, or a combination of these findings, which may require additional orthopedic measures (Figure 15E,F (Fig. 15)).

#### 5. Palliative treatment

**Figure 16:**
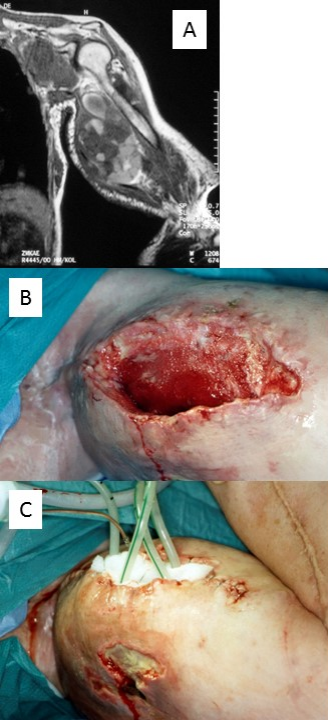
Palliative treatment for exulcerative malignant peripheral nerve sheath tumor of the upper extremity in an NF1 patient. A. Local extension in metastatic disease. B. Cavity of upper arm after removal of necrotic tissues. C. Application of suction drainage for vacuum treatment of wounds.

MPNST of the upper limb is a rare and life-threatening complication known in patients with and without NF1 [41], [42], [43]. MPNST contribute significantly to reduced life expectancy in NF1 [44]. However, the number of MPNST arising in pre-existing PNF appears to be low [45]. Organ-preserving ablative surgery is rarely an option. Amputation of the affected limb appears to increase survival chances [46]. In some cases, local extent of tumors and greatly diminished life expectancy may lead the treating surgeon to decide against making major interventions (Figure 16 (Fig. 16)). However, palliative measures are limited. In a recent series of MPNST those arising in the upper extremity and trunk showed the highest local recurrence rate [43]. However, the proportion of neurofibroma patients in this study was low (8%) [43].

## Discussion

This report describes personal experiences and analytical data of treating PNST of various types (nodular/diffuse/complex) of the upper extremity and hand in NF1 patients. The composition of the study group differs from other studies on surgical treatment of PNST in NF1, because patients have sought help for both aesthetic and functional limitations related to nerve sheath tumor development of the upper extremity and hand. However, analysis of clinical data allows some conclusions about the treatment concept in the individual NF1 patient affected with PNF of this region.

### Exclusion of analysis for other upper extremity and hand diseases related to NF1 in this study

Beyond the aim of this study are treatments for other certain lesions of the upper extremity and hand that are known to be associated with NF1, in particular bony alterations, e.g. erosions/osteolysis [47], [48], skeletal deformity [49], joint instability associated with PNF [50], pseudarthrosis [51], [52], [53], [54], [55], osseous local hypertrophy [56], [57], [58], [59], differentiation disorders of the hand [60], [61] including anatomical variations of hand muscles [62] and traumatic muscle disease in this entity [63]. Indeed, bone, including that in the upper limb, can be affected both locally and entirely in NF1 [64]. Vascular lesions, in particular acute hemorrhage following vascular malformation in the upper extremity of patients affected with NF1, are a well-documented manifestation of the disease of this region but are also not included in this report [65], [66], [67].

The aim of the study was to determine more precisely which surgical measures can be performed in connection with a PNF of the upper limb in NF1 patients and which treatment results are to be expected. Furthermore, the analysis focused on whether certain sections of the limb had been more frequently affected and whether an increase in operative interventions was required in these regions.

### Results

The presented results show that surgical treatment of PNF of the upper limb and hand can be performed successfully. The surgical measures provide patients with some relief from their often unsightly altered body region in many cases. However, the type and extent of the tumor are the most important features determining the effectiveness of surgery.

Functional disturbances are not expected with surgery for superficial PNF and are rarely synonymous with nodular PNF. However, there are reports of functional disruption after resection of extended nodular PNF of the hand and upper limb in patients with NF1 [34]. The risk is related above all to PNF of motor nerves [23].

**Figure 17:**
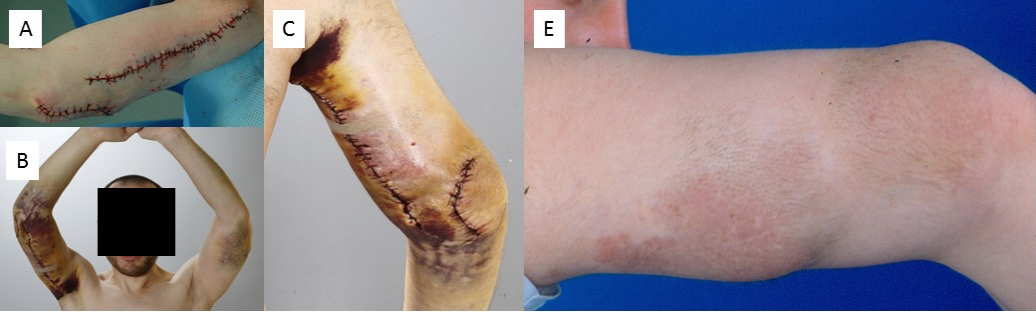
Patient with bilateral plexiform neurofibroma. A. Figure illustrates the situs of one side after surgery. B,C. Figures illustrate the hematoma that occurred several days later, after the patient had dispensed with the compression bandages. E. Figure illustrates patchy pigmentation of the right upper arm and sagging of the skin due to plexiform neurofibroma 5 years prior to present surgery of this region. Neither pigmentation nor hirsutism is coincident with the extent of the tumor.

**Figure 18:**
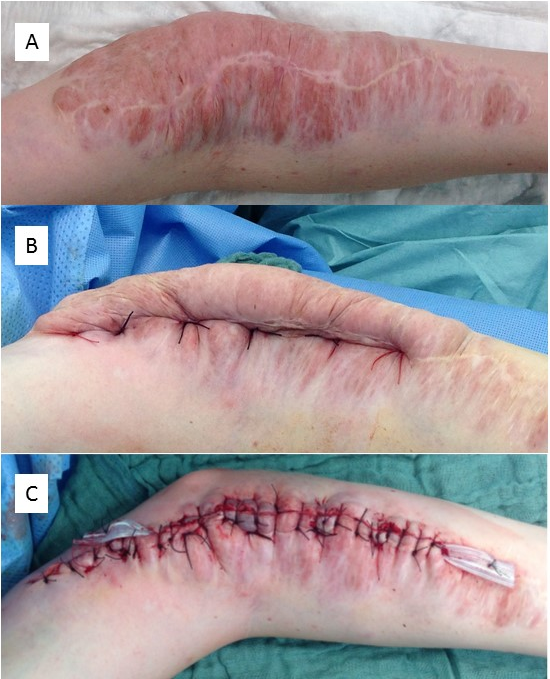
A–C. Deep ligatures are used to secure the vessels of the tumor volume to be resected from the blood flow.

The rate of complications was relatively low in the present study. However, extent of surgical intervention [18], complication rates [23] and success rates of PNST surgery rely strongly on the composition of the study group [18]. The patient’s willingness to cooperate is a factor that must not be underestimated in order to keep the complication rate low (Figure 17 (Fig. 17)). Deeply penetrating sutures below the tumor mass reduce bleeding (Figure 18 (Fig. 18)).

In larger studies on this subject, NF1 patients are usually in a minority [68]. Therefore, this discussion will first refer to some studies on the treatment of PNST published during the last 20 years, with special reference to the group of NF1 patients treated for PNST. In a further step, analysis of our own data is placed in the context of the literature.

### Comparison of results with other studies that include benign PNF of the upper limb and hand of NF1 patients

Artico et al. [69] studied 119 patients with benign PNST who were treated between 1980 and 1995. The number of NF1 patients was 25 (21%), with 5 patients affected with PNF. PNF originated from the median (n=3) or ulnar (n=2) nerve. However, most PNST of upper limb motor nerves were classified as neurofibroma (n=31). Pain was recorded in all patients with neurofibroma prior to surgery, irrespective of localization or type of tumor. The authors concluded that PNST surgery is unfavorable in the case of PNF. The frequency of local recurrence was estimated as 60% based on a small sample size.

Needle et al. [18] detailed their experiences in treatment and follow-up control of PNF in 121 children who were treated during a period of 20 years. The authors identified localization of PNF in the extremities to be a prognostic factor for longer intervals to progression. However, extent of surgical measure was also of prognostic significance. Near-total resection of the tumor allowed a median of more than a 10-year interval to tumor progression. This progression-free interval declined to 2 years if only biopsies had been performed. Permanent neurological complications were uncommon in this study (4.6%).

Kim et al. [21] analyzed patients treated for PNST at a single institution over a period of more than 30 years. Of a total sample size of 397 PNST the majority of tumors were benign (n=361; 91%). Neurofibroma was the most frequent diagnosis in benign PNST (n=237; 66%). NF1-associated neurofibroma was the second most common benign PNST (n=96; 41% of all neurofibromas). Diagnosis of neurofibroma dominated with respect to PNST of the upper extremity (n=78, i.e. 71% of 110 patients). A little less than half of the upper extremity neurofibromas arose in NF1 patients (n=33; 41%). Topography of resected neurofibroma was defined by the predominating tumorous nerve: ulnar nerve (n=15; 45%), median nerve (n=11; 33%) or radial nerve (n=7; 21%). Tumors were excised from all parts of the region (arm, elbow/forearm, wrist/hand). These authors detailed that in most cases no serious functional deficit was recorded even when tumor resection involved a major nerve. However, the authors admitted that resection of PNF was difficult. Results proved to be favorable in symptomatic tumors involving sensory nerves or branches, but “*complete removal generally was not possible without a loss of neurological functions … and even subtotal removal of a plexiform tumor led to some loss of function*” [21]. On the other hand, tumors involving superficial and less important nerves could be removed without serious functional loss. The authors recommended removal of large and firm PNF, irrespective of site, with explicit reference to the risk of malignant degeneration of these tumors. None of the MPNST in patients with NF1 in their series (n=13) arose in the upper extremity (but did arise in the brachial plexus, n=7) [21].

Onesti et al. [11] reported on surgical experience of benign PNST of the extremities. A total of 29 neurofibromas was noted in 17 patients. The authors referred to current diagnostic criteria to establish NF1 diagnosis. However, they did not expressly state whether NF1 patients were treated. Nevertheless, it is very likely that at least some patients in the study were NF1-affected. PNF were already noted during early childhood. The maximum dimension of tumors was 6 cm in diameter. A total of 12 neurofibromas had developed in the upper extremity. Tumor resection included epidermal and subcutaneous layers. Functional disorders were rarely noted after surgery (3 patients), of mild character and documented with electromyography. Recurrence rate was low (1 patient). No keloid or hypertrophic scar formation was noted in their study group. The authors emphasized the need to find an adequate compromise between the claim to the greatest possible tumor resection and the risk of functional damage in each individual case. In the same way, it is important to estimate the risk of tumor recurrence in subtotal tumor excision [11].

Levi et al. [70] reported on surgical treatment for 140 PNST in 132 patients who were subjected to surgery in a single institution (number of neurofibroma: 34, NF1-related neurofibroma: 22). NF1 patients were significantly younger than non-NF1 patients with tumors of same type. The most common presentations and symptoms in this subgroup were a space-occupying lesion (“mass”) and pain (82.4% and 73%, respectively), followed by numbness (26.5%) and weakness (23.5%). “Mass only” as a solitary finding was registered in about every fifth patient. Localization of neurofibroma in the upper extremity was seen in 29.4% (n=10) of the PNST upper extremity group. In 21.1% (n=4) of the MPNST group (n=19), the upper extremity gave rise to this tumor. However, 5 patients had NF1-related MPNST with no further specification for localization. The frequency of upper extremity MPNST was 6.06% of the total group with PNST in this body segment (n=66). The focus of this study was the impact of biopsy as a diagnostic indicator of higher risk of experiencing later nerve damage following definite surgery, and the impact of electrophysiological nerve monitoring to reduce surgical complications. Nerve monitoring was more frequently used in neurofibroma patients. The authors stated that their strategy has led to PNF tumors (nerves or their tumorous environment) with proven motor function never being sectioned and small tumor residues remaining in situ. Consequently, the authors restated their assertion that no tumor recurrence had occurred as a result of treatment, indicating that residual tumor had been left in 14.7% of neurofibroma procedures. Sensory deficiency was restricted *“to small peri-incisional areas or demarcated areas within the distal distribution of the peripheral nerve*” [70].

More recently, Montano et al. [22] analyzed the outcome of surgery for 173 PNST in 150 patients who were treated over a period of 31 years. Diagnosis of neurofibromatosis (not differentiated for type, termed “NF”) was a statistically significant prognostic factor for tumor recurrence. However, NF patients constituted a minority of their patient group (number of NF patients=13).

Desai [23] reported on the treatment of 442 patients with nerve sheath tumors of the neck and extremities. Of this large group of PNST, 52 (11.8%) showed features diagnostic for NF1. A total of 3 patients reported PNF. The average age of the NF1 group was 21.8 years. Topography of tumors of the NF1 group was not further specified. Tumor size alone was never accepted as a basis for surgery in asymptomatic patients. Indeed, preoperative neurogenic pain was noted in almost every patient in this study group (97%). Exclusively in the upper extremity, pain was noted in 170 patients. Motor weakness associated with upper extremity PNST was noted in 21 patients, predominantly for the ulnar nerve. Postoperatively, 32 patients with PNST of the upper extremity and hand were registered as having pain and 6 had a motor deficit (both predominantly in the ulnar nerve). These findings were not differentiated with regard to the genetic background of nerve sheath tumor development.

Total excision of neurofibroma was achieved in 24 patients. The excision of neurofibroma was assessed as gross total in 81 (18.2%) patients, all of them affected with PNST of the brachial plexus and extremities. The author emphasized that subtotal resection was performed in the rare case of tumors with multiple fascicle involvement and the risk of severe postoperative neurological deficit. This finding was explicitly found in connection with PNF. In no cases were intraoperative complications noted.

Guha et al. [71] described the experience of surgery for PNST in a Canadian hospital during a similarly long period of time to that presented in this study. The number of NF1 patients constituted 21.1% (n=37) of their whole study group (n=175). The total number of surgical procedures of the upper extremity and brachial plexus was 8 in the NF1 group. All surgically treated PNST of NF1 patients were localized in either supra- (n=7) or infraclavicular (n=1) parts of the brachial plexus. None of their patients was affected in the ulnar, radial or median nerve. This PNST distribution pattern for this region deviates strongly from the distribution in the reports of Kim et al. [21] and Artico et al. [69]. Significant intraoperative bleeding was noted in one case in the whole study group. This patient was affected with brachial plexus PNF. The rate of new sensory disturbances was low (3.7%) and they disappeared completely in time. An increase in scored pain was never noticed in any patient. Recurrence risk for NF1-associated neurofibroma was increased compared to other patients with PNST, and the effect of this genetic status persisted after elimination of PNF from the calculation. The general conclusion of the authors was that extent of resection was limited by the presence of PNF.

Assessment of this literature selection shows that the proportion of NF1 patients in the respective groups is very variable and distinction of the entity is not clearly made even in reports of the current literature [11], [22]. Furthermore, the initial findings for surgically treated PNST patients are also very variable: the presence of pain can be the dominant finding for the whole group [71], whereas in another study, aesthetic and functional impairments predominate [11]. In their collective statistics of the surgical treatment of PNST under the category “upper limb”, some authors state that they were performed exclusively or in the majority in the plexus brachialis [71], while others report in a similar form about interventions in this region which apply to the peripheral main motor nerves [21], [69]. In addition, the study groups differ considerably in the age structure of NF1-affected individuals [18], [21], [70], [71].

### Definition of PNF

It should be kept in mind that the term “neurofibroma” is defined morphologically [3]. Pathologists study and define PNST with the aid of diagnostic criteria that are generated by agreement of experts, and diagnostic experiences are passed on in a long line of colleagues [3]. In earlier times, the sometimes extraordinary and large tumor growth in patients who suffered from an unsightly looking disease that is now called NF1 led the treating physicians to coin neologisms to symbolize their visible findings. Prior to the existence of precise histological descriptions, the physical alterations of patients who we presently term as being affected with multiple CNF were described as “molluscum fibrosum” (in large numbers) [2]. The outdated term “Rankenneurom” is associated with Brun’s seminal descriptions of patients whose appearance would justify suspicion of NF1 and diffuse/invasive PNF [72], [73]. It is likely that the overlapping soft tissue tumors that follow the effect of gravity reminded him of sprouts and roots that grow along a wall. The metaphoric term is helpful in addressing the eventual volume of a tumor and the limits of surgery. However, the effect on deeper body parts cannot be estimated by this description. Furthermore, histology of these tumors can be – at least in part – identical to very flat and not necessarily extended tumors that are currently termed diffuse (cutaneous/dermal) neurofibroma or diffuse/plexiform neurofibroma. Localized and diffuse *cutaneous* neurofibromas are compared with *intraneural* neurofibromas. Intraneural neurofibromas are classified as localized or plexiform. Localized nodular neurofibromas can occur singly, but multiple occurrences in a defined region are not uncommon in NF1 patients. PNF are usually diagnosed when this type of solid tumor occurs in larger nerve fibers. The situation gets even more complicated when taking into account the phenotype of a completely enlarged anatomical unit, e.g. an enlarged upper limb. The metaphoric term for this phenotype is (localized) elephantiasis neuro(fibro)matosa [15]. However, this overgrowth does not necessarily affect both bone and soft tissue simultaneously. In the case of only partial soft tissue overgrowth, the term elephantiasis is also applicable. Further definition of the elephantiasis region points the clinician to the region of surgical interest. However, the metaphoric description does not give sufficient information about the internal composition of the tumor, the effect on structures localized inside the “lesion” or the histological tumor profile [3]. This difficulty in classifying tumors is evident in the fact that the present morphological term massive soft tissue neurofibroma [3] merely provides a somewhat more abstract description of the conditions which earlier have been described as elephantiasis [72], [73] (Table 5 (Tab. 5)). However, this currently used term can also be applied to body regions whose tumorous shape changes are not as obvious as they can be on the extremities. In massive soft tissue neurofibroma, all types of neurofibroma may occur [3].

Although it is reasonable to fall back on a diagnostic concept and terminology that classifies tumors with the aid of a magnification instrument applicable to prepared tissue samples, these descriptions are of secondary importance for surgical planning, so that the term “plexiform” in this application has a more metaphorical character for the description of a larger nerve sheath tumor (which may be associated with relevant alterations of adjacent organs such as vessels). Nevertheless, samples studied microscopically must be considered as representative of the entire specimen. It can be difficult to transfer the individual findings derived from a sample on a slide to the total resection specimen, in particular in large specimens [74].

Recently, classification of PNF in NF1 patients was proposed based on the analysis of tumors depicted using MRI [25], [26]. This classification uses a term derived from macroscopic and microscopic analysis for the description of larger tumors of the body, without a compulsory examination of the diagnosis by the pathologist. The classification addresses the growth types of these tumors with respect to the tissue layer affected (e.g. “superficial”, “deep”) and by analogy to simple objects (“nodular” or “pearl-like” aggregated lesion). Furthermore, the growth pattern of tumors is metaphorically linked to the suspected biological tumor behavior (“invasive” or “displacing”). These descriptions of tumors with reference to the MRI are extremely helpful in deciding whether to perform surgery and in surgical planning. Transfer of the histological term plexiform to describe the image of a suspected nerve sheath tumor provided by MRI (or other imaging techniques allowing visualization of soft tissues) is indeed obvious but problematic. In a strict sense, plexiform growth of a nerve sheath tumor seen in section images would refer to a space-occupying lesion that shows strand-like, intermingled components [3]. From the analogy to the histological concept, this description can only be applied to relatively large nodular PNST, whereas the plexiform growth pattern cannot be derived from the operative site or from the clinical imaging in the case of diffuse, often large-area neurofibromas. For these reasons, transformation of the currently genuinely histological concept “plexiform neurofibroma” is problematic for the description of clinical and radiological findings, especially in the case of extended tumors, because neither physical examination nor imaging can reliably conclude the tumor structure within the nerve or even its interaction with the environment. On the other hand, the tissue sample selected for histological investigation is assumed to be representative of the tumor differentiation throughout a possibly very large total volume. This inductive conclusion has genuine limits, which also correlate with the size of the resected tissue. Comparison of histological tumor classification and MRI-based classification in Table 5 (Tab. 5) shows that the classifications do not completely overlap. To this end, classification of the diagnostic criteria shows that both morphological and imaging classification can pass over individual entities because the same diagnostic criteria can be applied to different entities. Nevertheless, justified suspicion of PNF or malignant degeneration of a nerve sheath tumor into an MPNST can also be made on solitary, nodular-like lesions seen with MRI [26] or other imaging techniques [75].

The morphological basis of classification of surgical areas using dermatomes is based on the assumption that Schwann cells are the cells of origin for neurofibromas [76]. Nerve sheath tumors arising in peripheral nerves follow the course of these organs, at least at the beginning of tumor formation [3].

Although some statistically conspicuous clusters of adjacent dermatomes can be calculated in which surgical treatments of neurofibromas have been performed, the relationship of tumor expansion and corresponding topographically related dermatomes remains questionable. The topography of the tumors demonstrates that these associations predominantly are caused by:

1. a tumor growing on the border with neighboring dermatomes (i.e. the tumor develops in a (small) region bordering one or more dermatomes),
2. a tumor located inside a dermatome appearing to have grown randomly into one or several neighboring dermatomes (the association is produced by the (late) neoplastic behavior of the neurofibroma) or
3. the neurofibroma (almost) infiltrating the entire limb (so that the assignment according to dermatomes has no informational value and is obsolete).

**Figure 19:**
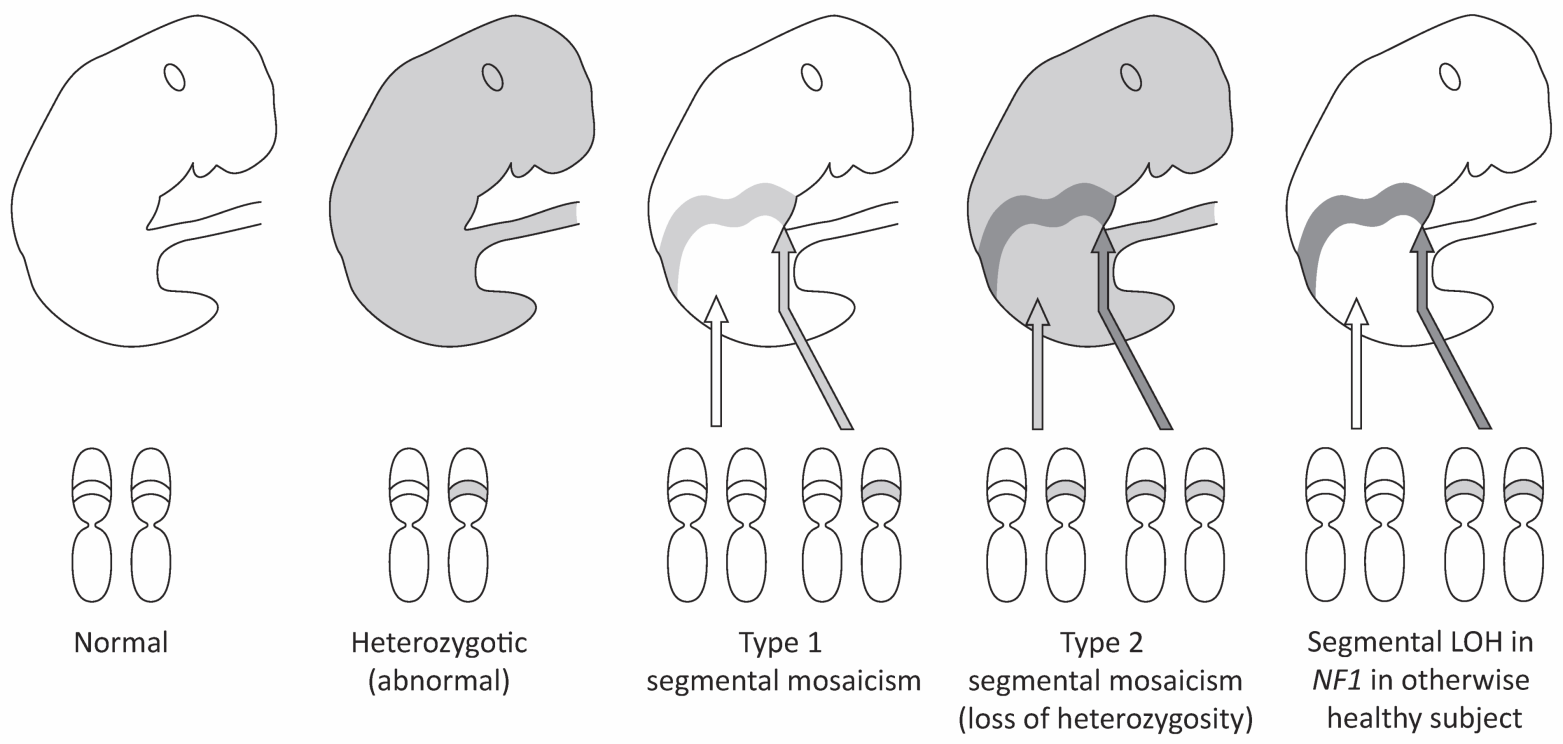
Schematic drawings of “segmental type 1 and type 2 mosaicism” in human skin according to Happle [77]. The prerequisite for the simplified presentation is a monogenic disease. The figures are slightly modified taking into account PNST in NF1. From left to right: in healthy individuals, both alleles relevant for the disease of interest are wildtype (white body). In an autosomal dominant hereditary disease such as NF1, an affected individual will carry the constitutional mutation in every single cell (gray body). Postzygotic mutation of one allele in a certain body region causes a heterozygous status in an otherwise healthy organism (light gray belt in white body): this status was also detected in individual cases for certain skin manifestations in *NF1* gene [101] and diagnosed as a “segmental type 1 manifestation” [77]. Tumors such as (plexiform) neurofibroma in an individual affected with NF1 are explained as the result of a second mutation at the NF1 gene in all tumor cells of the localized lesion: loss of heterozygosity (LOH, dark gray belt in light gray body). When this genetic event affects the skin, there is a “segmental type 2 manifestation” of this autosomal dominant trait [77]. The LOH appears to happen by chance [81], [83]. The time of mutation (ontogenesis, postnatal) is relevant for histological type of tumor and is crucial for tumor phenotype, e.g. on the upper limb. In rare cases, both alleles are mutated but these events are restricted to a body region or body segment. In this situation, a PNF can develop in a patient without meeting the clinical NF1 criteria when both mutated alleles of *NF1* have occurred in Schwann cells or Schwann cell progenitors (dark gray belt in white body). In principle, the above described distribution patterns of genetic events in a tumor suppressor gene causing an autosomal dominant inherited disease can all appear without skin involvement [79]. However, the pivotal involvement of the skin, bone and the nervous system in the genetic condition termed “NF1” are well established [1].

Happle has classified PNF as so-called type 2 segmental mosaicism in patients with proven NF1 (Figure 19 (Fig. 19)) [77]. The body segment affected by this mutation is not defined further in this classification. However, the term “segmental” must not exclusively refer to a skin region such as a dermatome, although Happle’s classification (and illustration) expressly refers to the integument (Figure 19 (Fig. 19)). A type 2 segmental mutation defines the local expression of the phenotype in an individual which already has a constitutional mutation [77]. It is conceivable that, in a very early developmental stage, the second allele mutates into a heterozygous carrier for the *NF1* gene. According to this model, loss of heterozygosity (LOH) [76] would occur, for example, in a Schwann cell or a precursor of this cell class and already control the development of the PNST before the formation of dermatomes. Using this model it could possibly be explained why the second mutation of the *NF1* gene can cause such different tumors and malformations (of skin) at different times of intrauterine development, i.e. the development of the PNF is independent of the outgrowth of the limb and associated development of the dermatomes.

On the other hand, according to other authors, the consistency of the spread of (plexiform) neurofibromas with the dermatomes of the skin must be accepted [72], [73], in particular wide agreement of PNF cutaneous extension with the course of the trigeminal nerve. The correlation of diagnostic pigmentary disorders of the skin with dermatomes has also been observed in individual cases of segmental NF1 [78]. These findings would be classified as type 1 segmental mutations of the skin [77]. However, no PNST should develop with a segmental type 1 manifestation of this autosomal dominant skin disease. In those patients where the upper extremity skin is affected, allocation of the spread pattern of the PNF’s cutaneous component to the dermatomes is only orientationally possible and cannot be detected in extended PNF/massive soft tissue neurofibroma according to our experience. Happle’s genetic model leaves open the question of how Schwann cells or their precursors intervene in the development of the organism. On the other hand, this model allows the influence of modifying genes on tumor development.

Further investigations have decoded the genetic mosaicism of NF1 [79]. However, the significance of these findings in the individual patient for PNF development and biological properties of this neoplasm has not yet been conclusively clarified.

The phenotype is also determined by postnatal growth of the tumors. In fact, postnatal tumor growth often accounts for the vast majority of the tumor volume that is to be surgically removed. Postnatal tumor growth can lead to the accidental infiltration of neighboring dermatomes, which is explained as the result of topographical relationships of the fully developed organism. However, as a rule, PNF are largely limited to the body area in which they have been diagnosed at birth or in the early infant phase. However, the extent of primary (connatal) tumor extension can escape the first diagnosis and only become noticeable in the course of the disease.

Therefore, the condition of the patient with regard to tumor manifestation and spread is determined by early genetic events as well as by further postpartum general development of the tumor. Possibly, the phenotype of a PNF addressed as “segmental type 2 mutation in NF1” can be understood as a (cutaneous) signal of locally misguided embryonic developmental control of the organism present at birth or in early childhood. A connective tissue cell of the nerve sheath with a defined and wide spectrum of mutations is causative for these developmental disorders. Later in life these lesions may be shaped both by physiological growth of the whole organism, interaction of the tumor with the surrounding tissue, and both metabolic and genetic changes within these failed nerve sheath cells. This view allows both perspectives on the PNF: hamartoma and neoplasm.

### PNF classification and tumor biology

Histological classification is also problematical with respect to tumor biology, because the same diagnosis (PNF) in different body regions presents a different risk to life. In general, PNF is regarded as a precancerous lesion [3]. However, MPNST of the head and neck region are rarely diagnosed in NF1 patients with facial PNF. Indeed, MPNST preferentially arise in the trunk and extremities. The risk of developing MPNST from PNF shows relation to the topography of tumors and histological type [33]. As a rule, nodular (intraneural, localized) PNF in deep body layers are more likely to develop into MPNST than more superficially located tumors [33]. Indeed, what is called a superficial (diffuse) PNF of even very large extent that shows unique characteristics on MRI [25], [26], [36], [37] appears to have only rarely the capacity to develop into MPNST [38].

**Figure 20:**
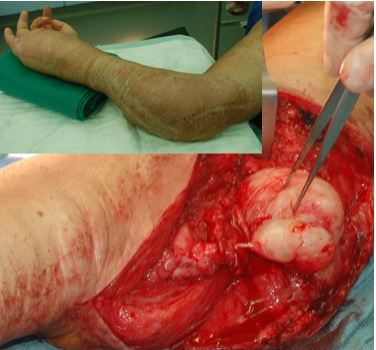
Massive soft tissue neurofibroma of the upper extremity. Several operations have already been performed to reduce the tumor. Within the soft tumor mass a tumor node has developed within the continuity of an enlarged nerve. The tumor was completely removed without functional impairment (insert: “elephantiasis” of this region is associated with local skeletal malformations).

On the other hand, it is well known that nodular PNST may be present inside a tumorous dewlap; the latter – with or without the nodule – would be classified in the fictitious case as a superficial diffuse (cutaneous) neurofibroma (optional of extraordinary size) covering a nodular PNF. This relationship was also addressed to the massive soft tissue neurofibroma [3]. In fact, in a particular case, there is hardly any difference between the two entities: an isolated (“localized”) nodular PNF or nodular PNF inside elephantiasis (Figure 20 (Fig. 20)).

Presently, genetic investigation of PNF is mainly used for scientific purposes. Second-hit mutations of the NF1 gene are the cause of both CNF and PNF [77], [80]. Type of tumor, in particular CNF vs. PNF, does not differ with reference to the type of mutation [81], [82]. However, large deletions have a higher risk of disintegrating cellular growth control and facilitating the development of malignant tumors [83], [84].

### PNF topography and surgical measures

In this study, there emerges no specific growth pattern of upper extremity and hand PNF in NF1 with respect to physical classification of tumor extent according to dermatomes. The skin region associated with the extent of a tumor was only seldom covered with the entire extent of the associated dermatome. Rather, the respective tumor often overlapped with border regions of neighboring dermatomes without following the distally or centrally directed extension of the dermatomes. In the area of elephantiasis-like tumor segments, classification was carried out, but in this case the cutaneous topography was only essential for planning the incision.

The incidental distribution of PNF within the region of interest of this study, i.e. hand and upper limb, should be expected in a disease with randomly distributed mutations [82]. There was no clear preference of body side in this study. Bilateral PNF was noted in upper extremity PNF, as already described in the literature [85].

However, all patients of the study were subjected to surgical intervention. Therefore, this patient group is strongly selected and very likely does not represent the expected distribution of hand and upper limb PNF in patients with NF1 in general [23]. Far more likely, the slightly unequal distribution of tumors categorized according to upper limb dermatomes reflects the individual need for surgical alleviation of signs and symptoms.

Wound healing in PNST surgery does not differ significantly between patients with and without neurofibromatosis [70], [86], [87]. In these studies, the wounds were primarily closed. However, the influence of swelling of the residual PNF on wound healing is not addressed in these studies. In the presented retrospective study, the influence of delayed wound healing on the duration of inpatient stay can only be determined approximately. Wound healing can be delayed by the reduction of large tumor masses and opening of large wound surfaces, especially in the case of partial tumor removal. The degree of successful wound healing was associated with the onset of epithelialization of the wound margins, which are tumor-invaded in diffuse neurofibroma. Extremely stretched scarring of the skin in the area of the wound occurs occasionally [24], [86].

Closure of defects by means of local tissue was efficient for wound management and contouring the body region [11]. However, the texture of the skin used for coverage may vary with respect to the grade of tumor invasion and also the age of the patient. Nevertheless, definitive wound closure is the primary objective of surgical care. Significantly delayed wound healing after resection of diffuse PNF must be expected when direct wound closure is not successful or is dispensed with [88]. PNF-invaded skin may be suitable for covering amputation defects [72].

Free skin transplants are suitable for defect coverage of small- to medium-sized lesions [85], [89]. There are only a few reports on the use of larger skin grafts for skin defect coverage in NF1 [90]. In extended resections of the extremities for the treatment of MPNST, if amputations can be avoided, the use of pedunculated or microvascular grafts is a valuable treatment option [39].

#### Complications

##### Bleeding

Bleeding is a serious complication in NF1 surgery [91], [92], [93], [94]. PNF vessels are the most frequent source of bleeding in soft tissue surgery for this disease. However, adequate conservative measures are usually sufficient to reduce the risks of bleeding (Figure 18 (Fig. 18)). Adaptation of the current size of the surgical field by means of successive tumor reduction and wound closure ensures the safe treatment of patients and considerably reduces intraoperative as well as postoperative blood loss [94]. The cooperation of the patient during postoperative follow-up is an essential part of a low complication rate (Figure 17 (Fig. 17)).

##### Neurological deficit

Permanent neurologic complications are uncommon in NF1 patients subjected to surgery [18]. Sensory deficits are usually limited to the region of surgical access to PNST and somewhat distal to it [82]. Significant motor deficit relies on the topography of PNF: reduction of PNF in motor nerves in a functional neuromuscular unit unavoidably affects signal transduction. Extent of surgery in these nerves depends on clinical parameters (pain, tumor biology).

### PNF progression

PNF of the upper extremity was a favorable prognostic factor concerning a longer interval to tumor progression [18]. Overall prognosis in childhood NF1 patients with PNF was assessed to be best in individuals affected in these body parts [18]. Repeated tumor reductions are valuable measures for aesthetic and functional rehabilitation [8]. In rare cases, amputation of a limb or parts thereof is unavoidable, e.g. in the case of extensive destruction of the bones or limb overgrowth, without detection of malignant degeneration [40], [59], [85]. In some cases, surgical measures can delay the indication of amputation in patients who are severely impaired by benign PNF of the upper extremity [95].

### PNF and MPNST

Reduced life expectancy in patients with NF1 depends to a large extent on the increased risk of developing malignant connective or other soft tissue neoplasms [44]. MPNST, formerly called neurofibrosarcomas [41], [42], are a well-known complication in NF1. About half of patients who develop MPNST will be affected with NF1 [68]. The risk for development of MPNST out of PNF is regarded as low [45]. However, the lifetime risk for developing MPNST in patients with this genetic background is estimated as about 10% [96]. Indeed, development of malignant tumors is regarded as the main cause for reduced life expectancy in patients with NF1. Mean lifetime of NF1 patients is expected to be about 8 years shorter than in the general population [97]. MPNST preferentially arise in the trunk and extremities [28]. The first presenting signs are usually a rapidly enlarging mass or unusual persistent pain [28], [41]. These nonspecific features of a neurogenic tumor complicate clinical differential diagnosis of benign tumors, especially in patients with a large tumor burden and chronic physical complaints.

Topography and localization of MPNST in NF1 do not differ from those found in sporadic cases [28], [68], [98], [99]. MPNST of cutaneous origin is rare [100]. Nevertheless, irrespective of the topography of tumor origin in this limb, the development of MPNST in the upper extremity is a rare diagnosis [9], [21], [22], [23], [28]. For example, Rogalski and Louis recorded 6 cases of neurofibrosarcoma arising in the upper extremity during an observation period of 38 years. All their patients were diagnosed as being affected with “neurofibromatosis” [41]. Given the relatively frequent finding of MPNST in NF1 and the rare occurrence of this entity in NF2, it is reasonable to assume that these authors have registered neurofibromatosis patients who would currently be diagnosed as having NF1. With reference to the probability that almost every second patient with MPNST suffers from NF1, differential diagnosis of NF1 is mandatory in any patient who develops MPNST. MPNST is a risk in NF1 and the goal of organ-preserving therapy (e.g. arm) [98] or radical surgery with safety margins [99] can be very difficult to implement in affected individuals [46].

## Conclusion

Plastic surgery can be very helpful in the treatment of upper limb and hand nerve sheath tumors in NF1 patients. Detailed imaging of the body region is a prerequisite for successful treatment. The complication rate can be kept low if the surgical targets are adapted to the tumor size and the wound conditions. In the case of malignant degeneration of a nerve sheath tumor, extended resection with a wide safety margins is the most important therapeutic measure.

## Notes

### Competing interests

The authors declare that they have no competing interests.

### Authorship

Both authors contributed equally to this publication.

### Acknowledgements

Expert pathological diagnosis of specimens was provided by Prof. C. Hagel, Institute of Neuropathology, UKE. The authors would like to thank Prof. Hagel for the valuable discussion on the terminology of peripheral nerve sheath tumors. Supervision of many patients documented in this study was provided by Prof. V. F. Mautner, Phakomatosen-Ambulanz, Department of Neurology, UKE. We thank G. Schön, Institute of Medical Biometry and Epidemiology, UKE, for his advice on statistical calculations.

### Conference presentation

The results of this study were presented in part in oral form on the occasion of the 20^th^ meeting of “Arbeitsgemeinschaft Neurofibromatosen”, Hamburg, 18^th^ and 19^th^ of November, 2016.

## Figures and Tables

**Table 1:**
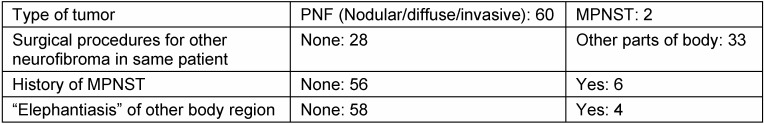
Some characteristics of patients with neurofibromatosis type 1 treated for plexiform neurofibroma of the upper limb and hand

**Table 2:**
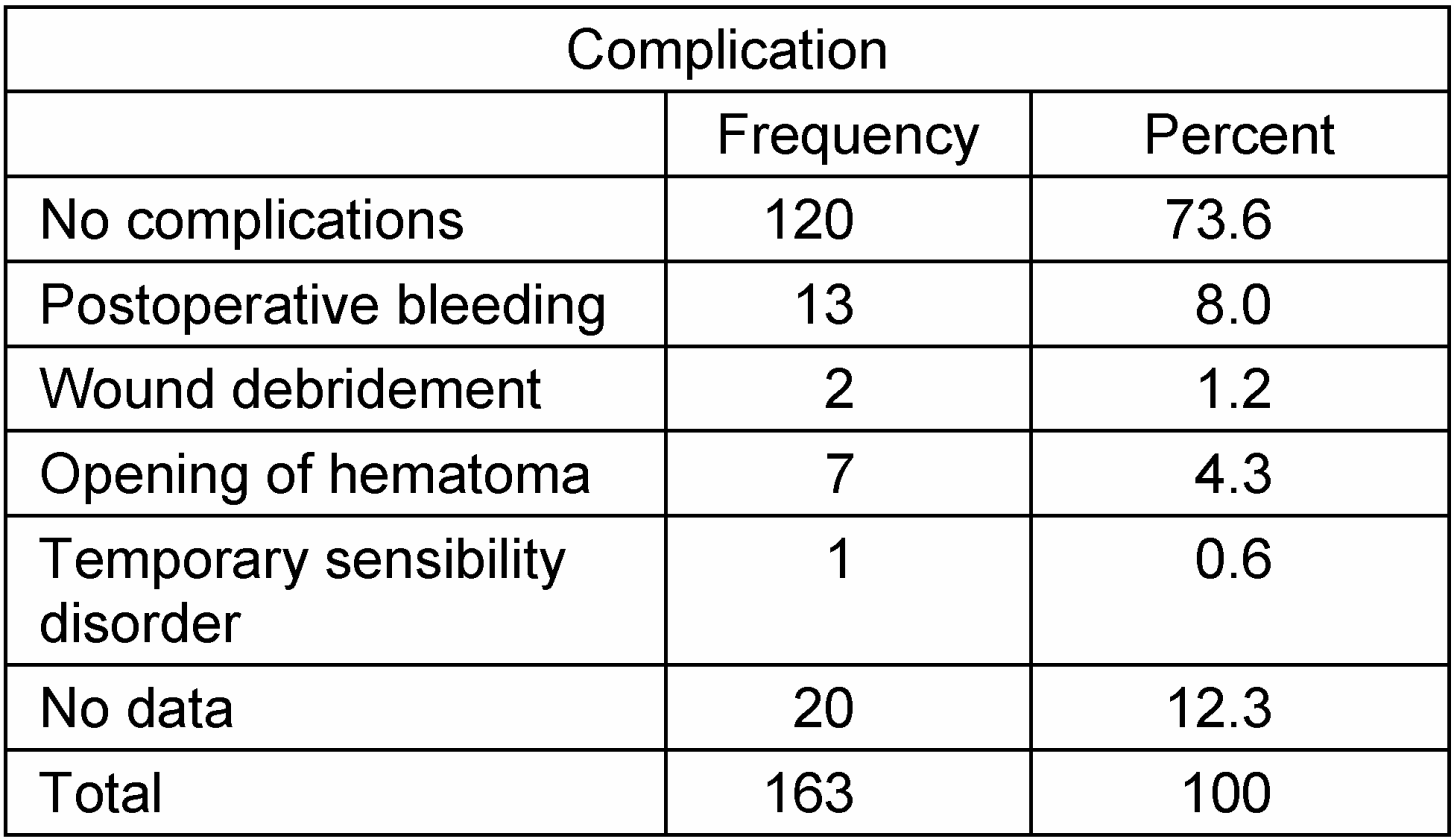
Complications during procedures to reduce or excise plexiform neurofibroma of the upper extremity and hand in patients with neurofibromatosis type 1

**Table 3:**
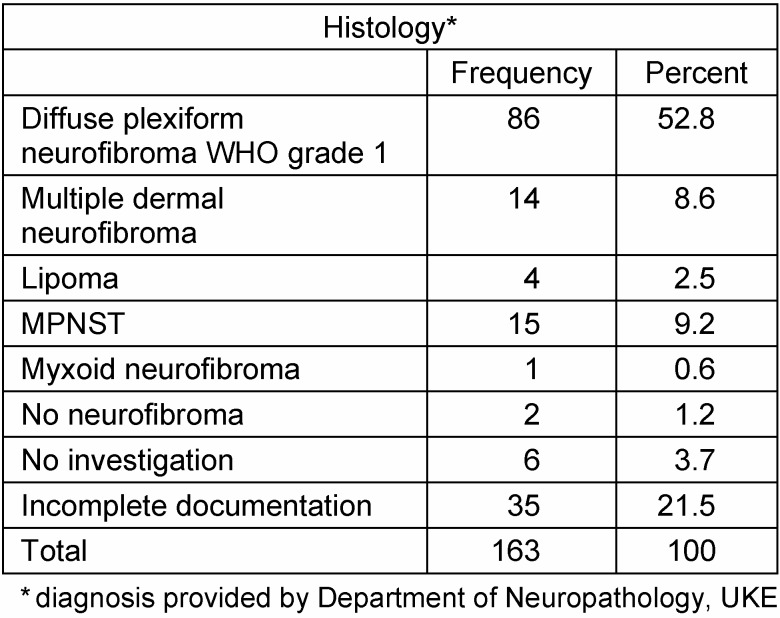
Histological findings in surgical procedures of 62 patients treated for nerve sheath tumors of the upper extremity and hand

**Table 4:**
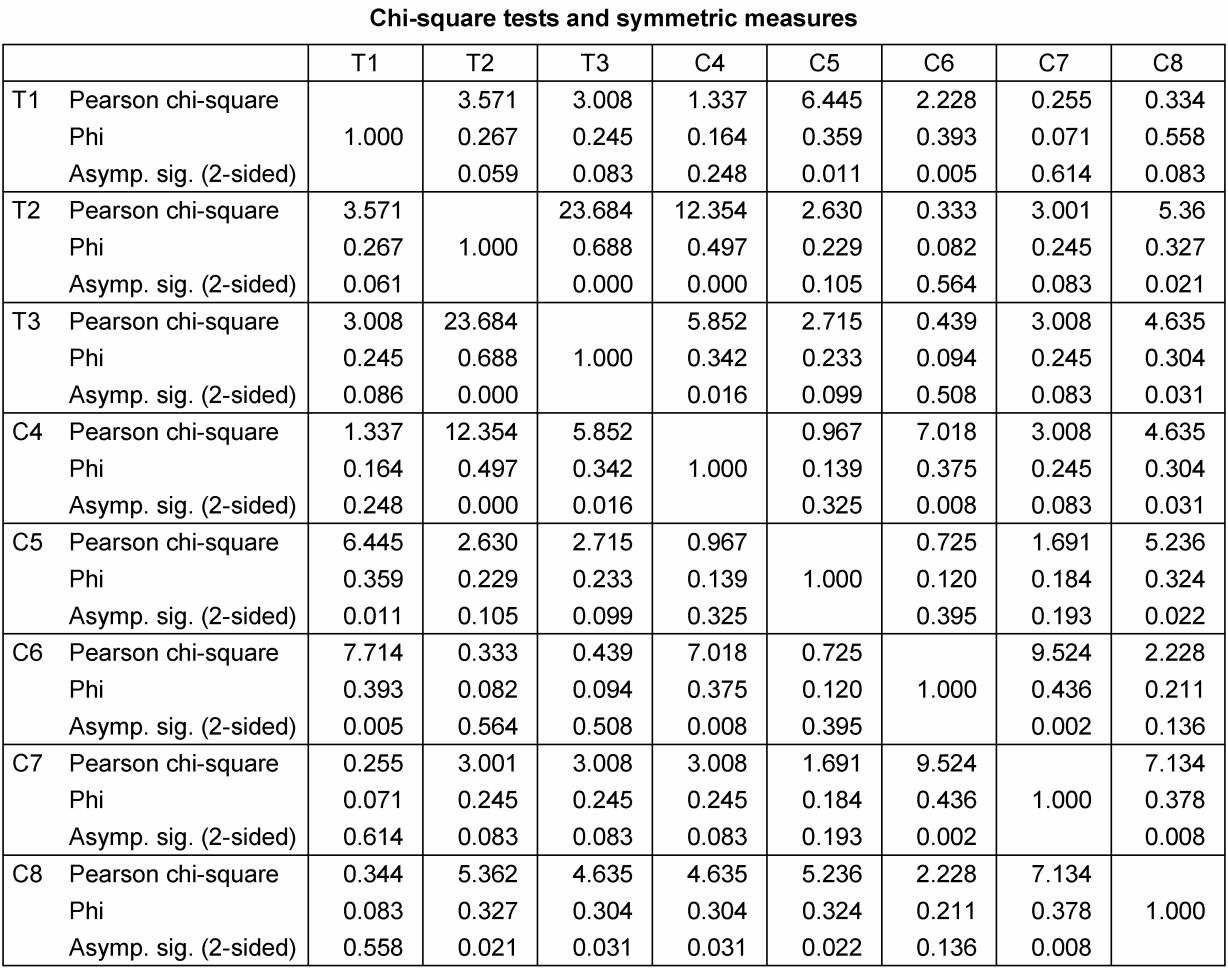
Correlations of tumor-affected dermatomes of the upper limb

**Table 5:**
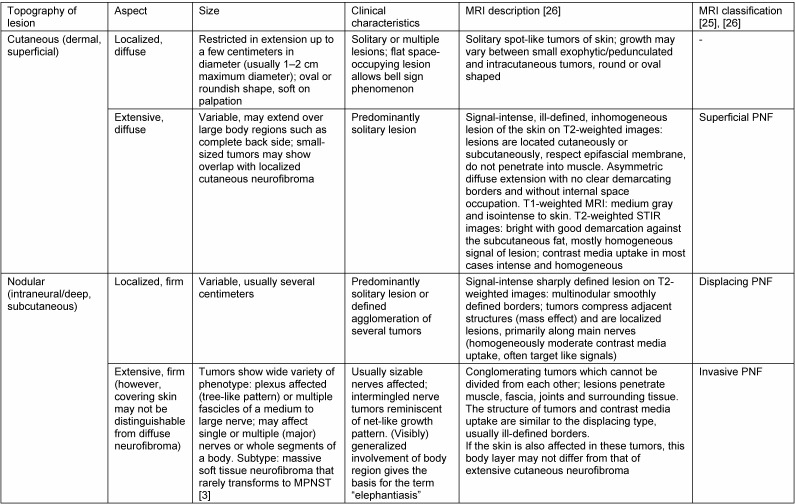
Characteristics of neurofibroma in neurofibromatosis type 1 [3]. Morphological classification represents a compromise between the visibly different tumor formations and the histological structure of nerve sheath tumors. Morphological classification is compared with MRI classification [25], [26].
